# Awareness of Hernias and Their Risk Factors Among Adults in the Western Region of Saudi Arabia

**DOI:** 10.7759/cureus.46994

**Published:** 2023-10-13

**Authors:** Elaf M Bahareth, Dhuha O Alquhra, Bushra M Alzhrani, Amjad S Alsulaihebi, Abdullah A Ali, Moath A Khayat, Hatim A Almaghrabi, Rani Alsairafi

**Affiliations:** 1 Medicine and Surgery, Umm Al-Qura University, Makkah, SAU; 2 Nephrology, AlNoor Specialist Hospital, Makkah, SAU; 3 Medicine and Surgery, Jazan University, Jazan, SAU; 4 Medical School, Umm Al-Qura University, Makkah, SAU; 5 General Surgery, Umm Al-Qura University, Makkah, SAU

**Keywords:** awareness of general population, saudi arabia, risk factors, awareness, hernia

## Abstract

Objective

To assess the awareness of hernia and its risk factors among adults in the western region of Saudi Arabia. Our hypothesis was that the studied population was in need of education regarding this matter.

Methods

This cross-sectional study was conducted in the western region of Saudi Arabia from November to December 2022, using a Google form. The generated questionnaire consisting of two parts was distributed electronically through social media platforms. The targeted population included adults from the western region of Saudi Arabia. The data were obtained using Microsoft Excel and analyzed using Statistical Package for Social Sciences (SPSS) v. 26 software (IBM Corp., Armonk, NY).

Results

A total of 1570 adults from the western region of Saudi Arabia were included in our study. We found that 13% of the participants had good overall knowledge about hernia, 31% had poor knowledge, and 56% had moderate knowledge. Alarmingly, 55% of the participants had a poor attitude, and 27% were evaluated as having poor practice. In addition, we found a significant association between people’s level of knowledge and multiple factors, including age (p-value_ _< 0.001), marital status (p-value< 0.001), and occupation (p-value< 0.001).

Conclusion

Based on our research, more than half of our population had a moderate level of knowledge regarding hernias including their risk factors and complications, with alarming levels of poor attitudes toward the disease. Healthcare workers were our participants' leading source of medical information, which directs us towards fully equipping them with the knowledge to educate the public effectively.

## Introduction

A hernia is the protrusion of an organ or part of an organ through a typical body wall [1]. To form a hernia, the abdominal wall structure membrane must lose its mechanical integrity, which inhibits the control of intra-abdominal forces during the Valsalva maneuver and the landing of the torso [2].

A hernia can be classified depending on its location. It can be classified as a midline hernia (epigastric and umbilical) or a lateral hernia (spigelian and lumbar). Hernias can also be classified based on their size. They can be either small (< 2 cm), medium (> 2-4 cm), or large (> 4 cm) [3]. A hernia in the inguinal region is the most common type, followed by a hernia in the femoral and umbilical areas [4].

Over 20 million hernias are estimated to occur worldwide each year [5]. Abdominal hernia repair is a common surgical procedure, with more than 990,000 procedures performed each year. Around 70,000 inguinal hernia surgeries were performed in the UK between 2001 and 2002, involving 0.14% of the population and resulting in more than 100,000 National Health Service (NHS) hospital bed days. Of these procedures, 62,969 repaired primary hernias. A cross-sectional study in the USA suggests that approximately 700,000 inguinal hernia surgeries were performed in 1993 [6].

A hernia itself is not harmful, but its complications are deadly, so any delay or failure to recognize a hernia may lead to devastating consequences [4]. Many hernia patients remain asymptomatic for long periods, and they experience pain, swelling, and heavy feelings in the abdomen at the time of presentation [7]. Therefore, having the wrong attitude toward hernia diagnosis and treatment can lead to many complications, such as strangulation, where the blood supply to the content of the hernia is compromised, and gangrene of the bowels; if not recognized and treated properly, mortality may be high as 30% due to passing of large hernia through small orifices and the adhesions that form between hernia content and the peritoneal lining of the sac [8].

Hernia is a common problem both locally and worldwide. A cross-sectional study conducted in 2021 reports that the prevalence of abdominal hernia was as high as 38.8% among the Saudi population [9]. In Riyadh, the capital of Saudi Arabia, a 2018 study reported a need for more knowledge about the predisposing factors of hernias among the Saudi population regardless of age and BMI. Many participants in the survey were able to connect hernias with heavy lifting, and less than 65% were aware that hernias could be associated with pregnancy and surgery. In contrast, less than half a percent of the study’s participants could connect hernias to other risk factors, such as asthma, enlarged prostate, smoking, diabetes, and chronic constipation [10].

Another study that took place in 2018 in the Al-Jouf region found that people had practical knowledge of the predisposing factors of hernias, regardless of their gender, age, and educational level. More than 50% of the participants from the study could associate heavy lifting, pregnancy, constipation, and previous surgery with hernias. In comparison, less than 50% of the chosen population could correlate smoking, enlarged prostate, and DM with hernias, clarifying that more studies are required to support their conclusion [11].

In Al-Qassim in 2020, a similar study was conducted, revealing that knowledge did not differ significantly by gender. However, in contrast to the previous two studies, knowledge levels appeared to rise among those aged 22 to 28 and to increase further based on their educational levels. The greatest risk factor that participants could identify was lifting heavy weights. Unfortunately, all participants did not correctly identify the remaining risk factors [12].

A study involving 1,119 Saudi citizens revealed a need for more awareness of the causes, risk factors, and treatment choices for inguinal hernias. About 79% of all participants in the study reported that they lacked a solid understanding of the causes, treatments, and prevention methods of hernias. Therefore, the study recommended further educational programs for the Saudi community to learn more about this widespread health issue [13].

More research has been done on hernia prevalence, risk factors, and awareness. For example, in Bangladesh in 2020, a prospective observational study of 100 patients admitted to a surgical department in a tertiary center found that most patients had low socioeconomic status, and their main risk factors were heavy lifting, smoking, aging, prostatism, and constipation and were affected by financial constraints, treatment by natural medicines, and fear of surgery due to lack of awareness and late presentations [14].

Despite this condition's common occurrence and clinical significance, limited data were available in Makkah. Thus, this study explores and assesses people’s awareness of hernias and their risk factors to improve mortality and morbidity rates through early detection, easy accessibility to medical facilities, and public health education.

## Materials and methods

Study design and setting

We employed a cross-sectional study using an online validated questionnaire prepared for distribution by Google Forms. The study was conducted among the general population of the western region of Saudi Arabia from November 2022 to December 2022.

Ethical considerations

We distributed our survey after obtaining ethical approval on 11/11/2022 from the Biomedical Research Ethics Committee of Umm Al-Qura University in Makkah, Saudi Arabia. The survey’s approval number is HAPO-02-K-012-2022-11-1241.

The subjects who participated in the survey were informed of their right to refuse participation and to end their involvement at any time. In addition, the study objectives, the methods that would be utilized to collect data, and the safety assurances were all explained to the subjects. This study maintained confidentiality and anonymity by refraining from disclosing the participants’ names on the questionnaire and research reports, keeping the collected data confidential, and refraining from revealing the subjects’ identities to anyone other than the current authors.

Eligibility criteria

Our study targeted the general population of Saudi Arabia’s western region, and we included males and females from the defined population over 18 years. However, physicians and people who refused to participate in the survey were excluded from the study.

Sample size

After determining our study population, we calculated the sample size using Open Epi version 3.0 (www.OpenEpi.com). Accordingly, the sample size required for this study was 385. We succeeded in including 1570 participants in our research.

Study tool

This study used a questionnaire that was designed and validated de novo following a review of the literature. The questionnaire was constructed based on the available literature and face validation was applied via the revision of two consultants. A pilot study was conducted, and the internal reliability test indicated that the questions accurately reflected a Cronbach's Alpha of 0.778. The questionnaire was generated by a Google form and distributed electronically via social media platforms, namely Twitter and WhatsApp. Informed consent was obtained from all participants to whom the study was thoroughly explained. The questionnaire included two parts. The first consisted of 11 questions concerning socio-demographic data (gender, age, nationality, residence, marital status, level of education, weight, height, occupation, and the source of information about hernia). The second part comprised 11 questions concerning hernia awareness and its risk factors.

Statistical analysis

A standard scoring method assessed the participants’ knowledge of hernias. One point was given for correct answers, and zero points were given for incorrect answers or participants’ inability to answer. Our tool had a total of 25 points; participants who answered at least two-thirds of the questions correctly were considered to have good knowledge about hernias, and participants who answered at least a third of the questions were regarded to have moderate knowledge. Lastly, participants who answered less than a third were considered to have poor knowledge. A similar method was used to calculate attitudes and practices toward hernias, dividing the participants into only two groups.

The data were entered in Microsoft Excel spreadsheets. Then, it was transferred into Statistical Package for Social Studies (SPSS) v.26 (IBM Corp., Armonk, NY) spreadsheets. The frequency was calculated for the categorical variables and the mean ± standard deviation for the continuous variables. The chi-square test was used to compare categorical variables. Univariate analysis was done to find associations between the participants’ level of awareness toward hernia and its risk factors and their gender, nationality, city of residence, marital status, level of education, occupation, source of medical information, and self-reported sufficiency of sources of medical information.

## Results

A total of 1570 adults from the western region of Saudi Arabia were included in our study, with a nearly equal distribution of male and female responders. The mean age was 31, with a standard deviation of 12. The average BMI of the participants was 25.8, with a standard deviation of 6.5. Participants from different cities in the region and various socioeconomic statuses were included. Most of the participants relied on healthcare workers and internet articles as their primary source of medical information; only 31% of participants reported believing their information source was sufficient. More details regarding socio-demographic characteristics are provided in Table 1.

**Table 1:** Socio-demographic characteristics of study participants

Characteristics		N	%
Gender	Male	700	45%
	Female	870	55%
Nationality	Saudi	1458	93%
	Non-Saudi	112	7%
City of residence	Makkah	565	36%
	Jeddah	533	34%
	Medina	191	12%
	Taif	128	8%
	Yanbu	127	8%
	Qunfudhah	26	2%
Marital status	Married	619	39.50%
	Divorced	47	3%
	Widow/er	22	1.50%
	Single	882	56%
Educational level	Elementary school	5	0.30%
	Intermediate school	20	1%
	High school	406	26%
	Bachelors	1048	66.70%
	Higher studies	91	6%
Work	Unemployed	728	47%
	Freelancer	84	5%
	Retired	80	5%
	Healthcare worker (All except physicians)	235	15%
	Fieldworker	267	17%
	Office worker	176	11%
Primary source of medical information	Family and friends	216	14%
	Internet articles	520	33%
	Social media	265	17%
	Health care workers	556	35%
	Health promotion campaigns	13	1%
Self-reported sufficiency of the previous source	sufficient	482	31%
	Not sufficient	650	41%
	Almost sufficient	438	28%

**Figure 1:**
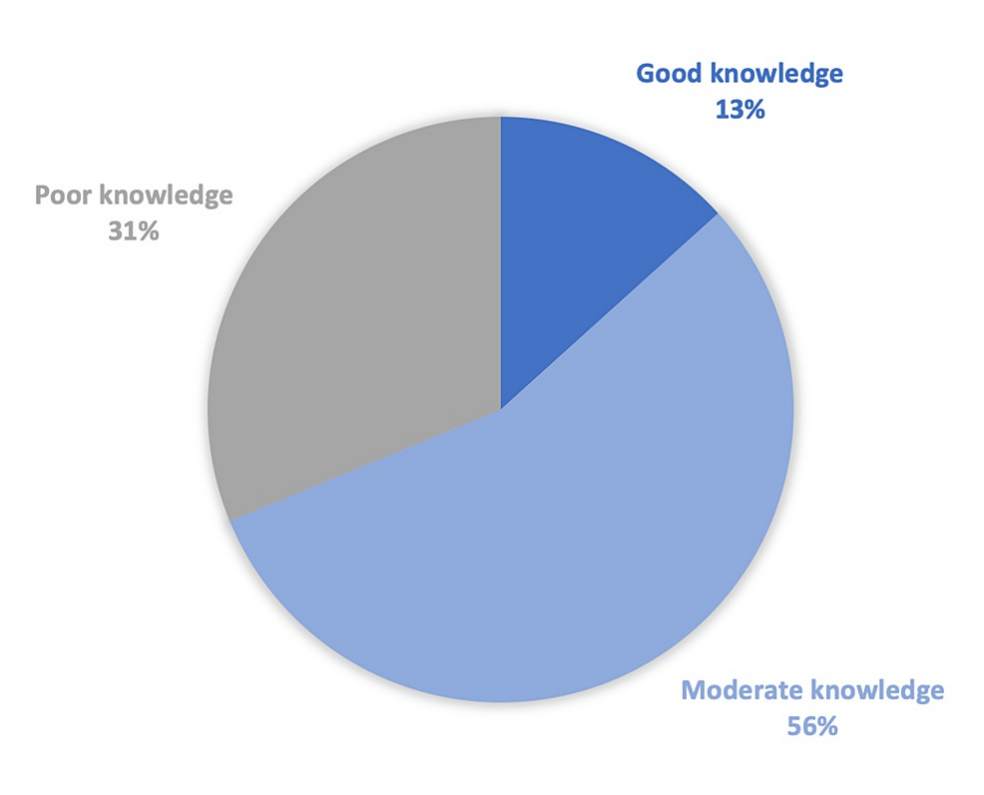
Knowledge assessment

In Figure 1, more than half of the responders scored moderate knowledge. Some of the main highlights reflected by this percentage are seen in questions regarding the definition of hernia; nearly half of the participants thought a hernia was a rupture in the muscles of the abdomen. More than 85% of participants did not identify smoking, prostate hyperplasia, steroid use, or significant weight loss as hernia causes. Table 2 contains a breakdown of answers regarding knowledge.

**Table 2:** Breakdown of answers

Question	Choices		Frequency	Percentage
Q1. In your opinion, which of the following best describes hernia?	Protrusion of all or a part of any intraabdominal structure due to any congenital, acquired, or iatrogenic defect in the abnormal wall		562	36%
	Dilated veins within the anal canal that may abnormally enlarge, and protrude		194	12%
	Tear or rupture of a part of the abdominal muscles		731	47%
	A cut wound in the abdomen		82	5%
Q2. If you noticed having the previous description, how long would it take you to seek help?	Immediately		773	49%
	Up to 7 days		543	35%
	Up to 14 days		128	8%
	More than 14 days		125	8%
Q3. Are there many types of hernias?	Yes, there are many types		1260	80%
	No, there is only one type		309	20%
Q4. What are the causes of hernia in your opinion? (Each of these causes was asked individually)	Abdominal muscle weakness	Yes	1064	68%
		No	506	32%
	Weightlifting sports	Yes	952	61%
		No	618	39%
	Pregnancy and delivery	Yes	830	53%
		No	740	47%
	Abdominal and bariatric surgery	Yes	592	38%
		No	978	62%
	Congenital	Yes	669	43%
		No	901	57%
	Obesity	Yes	828	53%
		No	742	47%
	Chronic constipation	Yes	480	31%
		No	1090	69%
	Chronic cough	Yes	515	33%
		No	1055	67%
	Smoking	Yes	195	12%
		No	1375	88%
	Prostate hyperplasia	Yes	223	14%
		No	1347	86%
	Steroid use	Yes	133	9%
		No	1437	91%
	Significant weight loss	Yes	171	11%
		No	1399	89%
Q5. Which of these do you consider as a warning sign of hernia? (Each of these causes was asked individually)	Inability to reduce the hernia	Yes	855	55%
		No	715	45%
	Signs of bowel obstruction (nausea, vomiting, abdominal pain and distension, constipation)	Yes	888	57%
		No	682	43%
	Sudden, severe pain	Yes	1005	64%
		No	565	36%
	Signs of sepsis and shock (Altered mental state, weak or rapid heartbeat, etc.)	Yes	544	35%
		No	1026	65%
	Change of overlying skin (redness, hotness, etc.)	Yes	494	32%
		No	1076	68%
Q6. If you had one of the previous symptoms, how long would it take you to seek help?	Immediately		1104	70%
	Up to 7 days		336	22%
	Up to 14 days		78	5%
	More than 14 days		51	3%
Q7. Regarding the previous question, which of the following would you ask for help?	Professional medical attention		1397	89%
	Herbs		44	3%
	Traditional therapy (e.g., cautery)		36	2%
	Family and friends		93	6%
Q8. Do you think immediate surgery is indicated for all cases of hernia?	Yes		706	45%
	No		863	55%
Q9. Do you think hernia is a curable disease?	Yes		1464	93%
	No		105	7%
Q10. How common do you think recurrence of hernia is?	High recurrence		261	17%
	Moderate recurrence		1095	70%
	No recurrence		214	13%
Q11. Which of the following do you think is a complication of hernia? (Each of these complications was asked individually)	Strangulation and incarceration	Yes	1058	67%
		No	512	33%
	Bowel obstruction and ischemia	Yes	803	51%
		No	767	49%
	Perforation of the abdomen	Yes	586	37%
		No	984	63%
	Shock	Yes	594	38%
		No	976	62%

**Figure 2:**
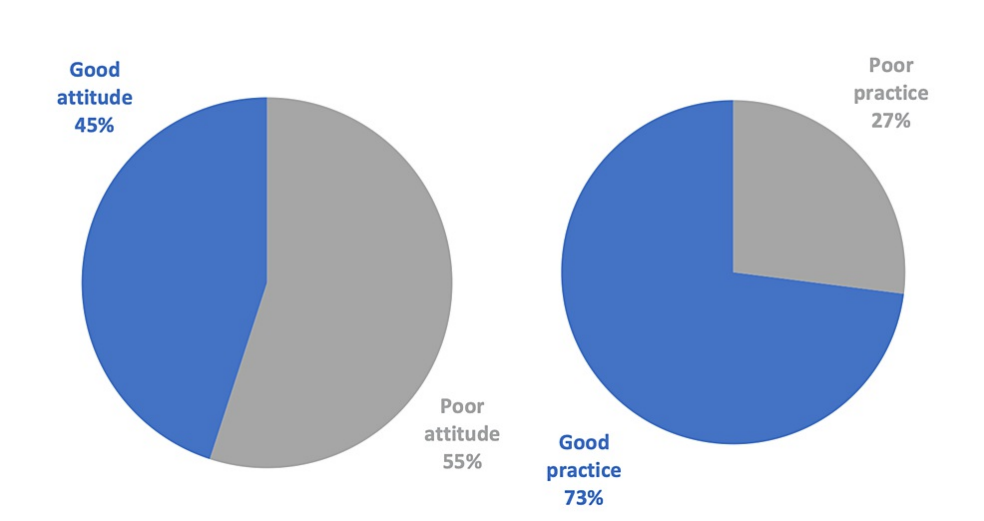
Attitude and practice assessment

In Figure 2, the level of practice was consistent with the knowledge level; however, attitude levels were dramatically lower.

Our data analysis demonstrates a significant association between the level of knowledge and gender; female participants had a slightly higher level of knowledge. The city of residence was also significantly associated, with Medina and Taif having the highest knowledge among region residents. Marital status was significant, with single participants having a higher level of knowledge than other groups. The primary source of medical information was also significantly associated, with participants who relied on their family and friends having the lowest level of knowledge among other groups; other associations are found in Table 3.

**Table 3:** The relationship between the socio-demographic characteristics of participants and their level of knowledge

Characteristic	Level of knowledge						P-value
	Good knowledge		Moderate knowledge		Poor knowledge		
	Frequency	Percentage	Frequency	Percentage	Frequency	Percentage	
Gender							
Male	103	49%	329	38%	268	55%	<0.001*
Female	106	51%	542	62%	222	45%	
Nationality							
Saudi	197	94%	808	93%	453	92%	0.69
Non-Saudi	12	6%	63	7%	37	8%	
City of Residence							
Makkah	71	34%	319	36%	175	36%	<0.001*
Jeddah	54	26%	296	34%	183	37%	
Medina	43	21%	102	12%	46	9%	
Taif	29	14%	64	7%	35	7%	
Yanbu	9	4%	75	9%	43	9%	
Qunfudhah	3	1%	15	2%	8	2%	
Marital Status							
Married	56	27%	347	40%	216	44%	<0.001*
Divorced	11	5%	22	3%	14	3%	
Widow/er	1	1%	11	1%	10	2%	
Single	141	67%	491	56%	250	51%	
Level of Education							
Elementary school	1	0.5%	1	0.1%	3	1%	0.142
Intermediate school	1	0.5%	10	1%	9	2%	
High school	68	33%	224	25.9%	114	23%	
Bachelor	130	62%	587	67%	331	67%	
Higher studies	9	4%	49	6%	33	7%	
Occupation							
Unemployed	78	37%	434	50%	216	44%	<0.001*
Freelancer	9	4%	46	5%	29	6%	
Retired	7	3%	40	5%	33	7%	
Healthcare workers (All except physicians)	79	38%	123	14%	33	7%	
Fieldworker	18	9%	129	15%	120	24%	
Office worker	18	9%	99	11%	59	12%	
Primary source of medical information							
Family and friends	14	7%	95	11%	107	22%	<0.001*
Internet articles	79	38%	315	36%	126	26%	
Social media	31	15%	128	15%	106	21%	
Health care workers	82	39%	323	37%	151	31%	
Health promotion campaigns	3	1%	10	1%	0	0%	
Self-reported sufficiency of previous source							
Sufficient	82	39%	232	27%	168	34%	0.001*
Not sufficient	70	34%	382	44%	198	41%	
Almost sufficient	57	27%	257	29%	124	25%	

## Discussion

Our study aimed to assess awareness of hernias and their risk factors among adults in the Western region of Saudi Arabia.

The study found that there was not a considerable difference in understanding about hernia between different genders. This finding is also consistent with many studies in the literature, including those conducted in Al Qassim, Riyadh, and Al-Jouf [10-13]. Therefore, in general, it is not necessary to take gender into account when attempting to raise public knowledge and awareness of hernia risk factors.

Our study found that people aged 21 to 30 were more knowledgeable and aware of hernia risk factors than others. We believe this is because people in this age group are most frequently exposed to information about hernias in school. This result was consistent with two independent studies in Al Qassim and Riyadh, which discovered that this age group seemed to have higher knowledge about hernias than other age groups [12,13]. Therefore, we may conclude that it is more important to concentrate on spreading knowledge of hernias among populations under the age of 21 and those over 30 because they have insufficient information about hernias.

In addition, participants with higher levels of education appeared to know more about hernias since we discovered that those with bachelor’s degrees statistically showed more knowledge about the risk factors for hernias and various elements of the condition. This is consistent with several previous studies conducted in Al Qassim (2020), Riyadh (2020), and Al-Jouf (2018), as well as a 2017 study conducted in Al Qassim to assess people’s knowledge of diabetic mellitus, which found a link between people’s educational level and a better level of knowledge about diabetic mellitus [11-13,15].

Several studies state that obesity is a well-known risk factor for developing hernias. Thus, we decided to include BMI in our research questionnaire [16]. We discovered that the participants had a BMI average of 25.8, and 56% had moderate knowledge of hernias regardless of their BMI. This is consistent with a 2018 study that focused on assessing the awareness of risk factors of hernias among adults in Riyadh [9].

Our analysis revealed that unemployed participants had higher awareness levels of hernia risk factors than healthcare professionals (excluding physicians), which may be related to the fact that most of our sample was composed of unemployed people (728 out of 1570). A 2020 study on the Saudi population reported a similar finding that aimed to assess people’s awareness and knowledge of the causes, risk factors, and management of inguinal hernia. Most of this study’s sample was unemployed (675 out of 1119) [8].

Interestingly, we noted a connection between good knowledge of hernias and marital status and discovered that nearly half of the study’s single participants, which made up 56.1% of the total study population of 15.9% had good knowledge about hernias. However, a different study conducted in Jeddah that evaluated breast cancer awareness revealed that single female participants knew more about breast cancer than other participants [17].

Although the sample showed good knowledge of hernias, this differed from their attitudes. Since healthcare workers are our participants' leading source of medical information, we recommend that medical school curricula consider this when topics related to hernias are taught to medical students. This will allow them to understand the societal level of knowledge about hernias as they deliver scientific material to the public.

This is the first study to assess adults’ knowledge about hernias and their risk factors and the first study to connect the element of BMI to hernia knowledge in Saudi Arabia’s western region. Although our research had the benefit of using primary data, which resulted in less information loss and a large sample, it was restrained because it was a cross-sectional study that could be biased. Therefore, we strongly advocate performing additional in-depth research with larger samples, which include individuals from various locations in Saudi Arabia, to measure how knowledgeable Saudi Arabian citizens are regarding abdominal hernias and their associated risk factors.

## Conclusions

Based on our research, more than half of our population had a moderate level of knowledge regarding hernias including their risk factors and complications, with alarming levels of attitudes toward the disease. Healthcare workers were our participants' leading source of medical information, which directs us towards fully equipping them with the knowledge to educate the public effectively. 
